# Sleeping sickness in the historical focus of forested Guinea: update using a geographically based method

**DOI:** 10.1051/parasite/2019061

**Published:** 2019-10-10

**Authors:** Fabrice Courtin, Oumou Camara, Mamadou Camara, Moïse Kagbadouno, Bruno Bucheton, Philippe Solano, Vincent Jamonneau

**Affiliations:** 1 Institut de Recherche pour le Développement (IRD), UMR 177 IRD-CIRAD INTERTRYP, Institut Pierre Richet/Institut National de Santé Publique Bouaké Côte d’Ivoire; 2 Programme National de Lutte contre la THA, Ministère de la Santé Conakry Guinea; 3 Institut de Recherche pour le Développement (IRD), UMR 177 IRD-CIRAD INTERTRYP, Programme National de Lutte contre la THA Conakry Guinea; 4 Institut de Recherche pour le Développement (IRD), UMR 177 IRD-CIRAD INTERTRYP Montpellier France

**Keywords:** Sleeping sickness, *Trypanosoma brucei gambiense*, Guinea, Geography, Risk, Elimination

## Abstract

In 2017, 1447 new cases of Human African Trypanosomiasis (HAT) were reported, which reflects considerable progress towards the World Health Organisation’s target of eliminating HAT as a public health problem by 2020. However, current epidemiological data are still lacking for a number of areas, including historical HAT foci. In order to update the HAT situation in the historical focus of forested Guinea, we implemented a geographically based methodology: Identification of Villages at Risk (IVR). The methodology is based on three sequential steps: Desk-based IVR (IVR-D), which selects villages at risk of HAT on the basis of HAT archives and geographical items; Field-based IVR (IVR-F), which consists in collecting additional epidemiological and geographical information in the field in villages at risk; and to be Medically surveyed IVR (IVR-M), a field data analysis through a Geographic Information System (GIS), to compile a list of the villages most at risk of HAT, suitable to guide active screening and passive surveillance. In an area of 2385 km^2^ with 1420,530 inhabitants distributed in 1884 settlements, 14 villages with a population of 11,236 inhabitants were identified as most at risk of HAT and selected for active screening. Although no HAT cases could be confirmed, subjects that had come into contact with *Trypanosoma brucei gambiense* were identified and two sentinel sites were chosen to implement passive surveillance. IVR, which could be applied to any *gambiense* areas where the situation needs to be clarified, could help to reach the objective of HAT elimination.

## Abstract (fr)

En 2017, 1447 nouveaux cas de Trypanosomiase Humaine Africaine (THA) ont été rapportés, ce qui constitue une avancée importante pour atteindre l’objectif affiché par l’OMS d’éliminer la THA comme problème de santé publique d’ici 2020. Cependant, il existe toujours un manque d’informations épidémiologiques dans certaines zones, incluant des foyers historiques de THA. Afin d’actualiser la situation de la THA dans le foyer historique de Guinée forestière, nous avons appliqué une méthode géographique : l’Identification des Villages à Risque (IVR). La méthode s’effectue en 3 étapes successives : l’identification des villages à risque au bureau (IVR-D), qui sélectionne des villages à risque de THA sur la base d’archives de la THA et d’éléments géographiques ; l’identification des villages à risque sur le terrain (IVR-F), qui consiste à collecter des données épidémiologiques et géographiques des villages à risque sur le terrain ; l’identification des villages à risque à prospecter (IVR-M), une analyse des données de terrain, à travers un système d’information géographique, visant à dresser une liste de villages les plus à risque de THA, qui permettront d’orienter le dépistage actif et la surveillance passive. Dans une aire de 2385 km^2^, avec 1 420 530 habitants distribués dans 1884 peuplements, 14 villages d’une population de 11 236 habitants ont été identifiés comme les plus à risque de THA et sélectionnés pour un dépistage actif. Bien qu’aucun cas de THA n’ait été confirmé, des individus qui sont entrés en contact avec *Trypanosoma brucei gambiense* ont été identifiés et 2 sites sentinelles ont été retenus pour la surveillance passive. IVR, qui pourrait être appliquée dans n’importe quelle zone à *gambiense* où la situation nécessite d’être clarifiée, pourrait aider à atteindre l’objectif d’élimination de la THA.

## Introduction

Human African Trypanosomiasis (HAT), or sleeping sickness, is a lethal disease caused by the transmission of trypanosomes to humans by tsetse flies in Sub-Saharan Africa. In the first half of the 20th century, HAT rendered several regions uninhabitable, necessitating the creation of specific services to control the disease [6]. The chronic form of HAT occurring in West and Central Africa is assumed to be mainly an anthroponosis caused by *Trypanosoma brucei gambiense* (*Tbg*), and its control has primarily been based on active screening and treatment [13]. This active screening has typically been performed by mobile teams that move from one village to the next, with the aim of screening entire populations in HAT endemic areas.

After several substantial HAT epidemics in the early 20th century, the situation was considered to be under control by the 1960s in most African countries, such that the professionals in charge of HAT control referred to it as “*trypanosomiase résiduelle*” (residual trypanosomiasis) [18]. However, this early sense of victory was probably responsible in part for a decrease in control activities. This occurred in a context of population growth, economic development, landscape change and political instability, allowing the re-emergence of HAT during the 1970s in Central [17], Eastern [9] and Western Africa [4]. In 1998, the World Health Organisation (WHO) reported 30,000 cases of HAT and an estimated total of 300,000 infected individuals [22].

The situation improved gradually after a new phase of intense medical control. In 2009, the number of new cases reported by the WHO dropped below 10,000 for the first time in 50 years (representing a 63% decrease since 2000), concurrent with an increase in the number of people screened [20]. Recently, the WHO confirmed its target of eliminating HAT as a public health problem by 2020, defined as a 90% reduction in the total area at risk reporting ≥1 case/10,000 people/year (based on the 2004 WHO baseline levels), and less than 2000 new cases reported annually at the continental level. In addition, interruption of transmission has been set as an objective for 2030 [23]. In 2017, 1447 new HAT cases were reported across the African continent [24]. The current situation is thus similar to the trend in the 1960s, when the number of new HAT cases was less than 5000 for the whole continent [8]. To reach the 2020 and 2030 elimination objectives, it is crucial to learn from history and develop tools and strategies adapted to the current low prevalence of HAT.

Within this context, there are still historical HAT foci or regions that are favourable to its re-emergence, where medical surveillance has not been implemented for a long time (or was never implemented) and where the current epidemiological status is unknown or remains unclear [3]. Most of these areas have been classified by the WHO as “foci requiring further investigations to assess intensity of transmission” [23].

**Figure 1:**
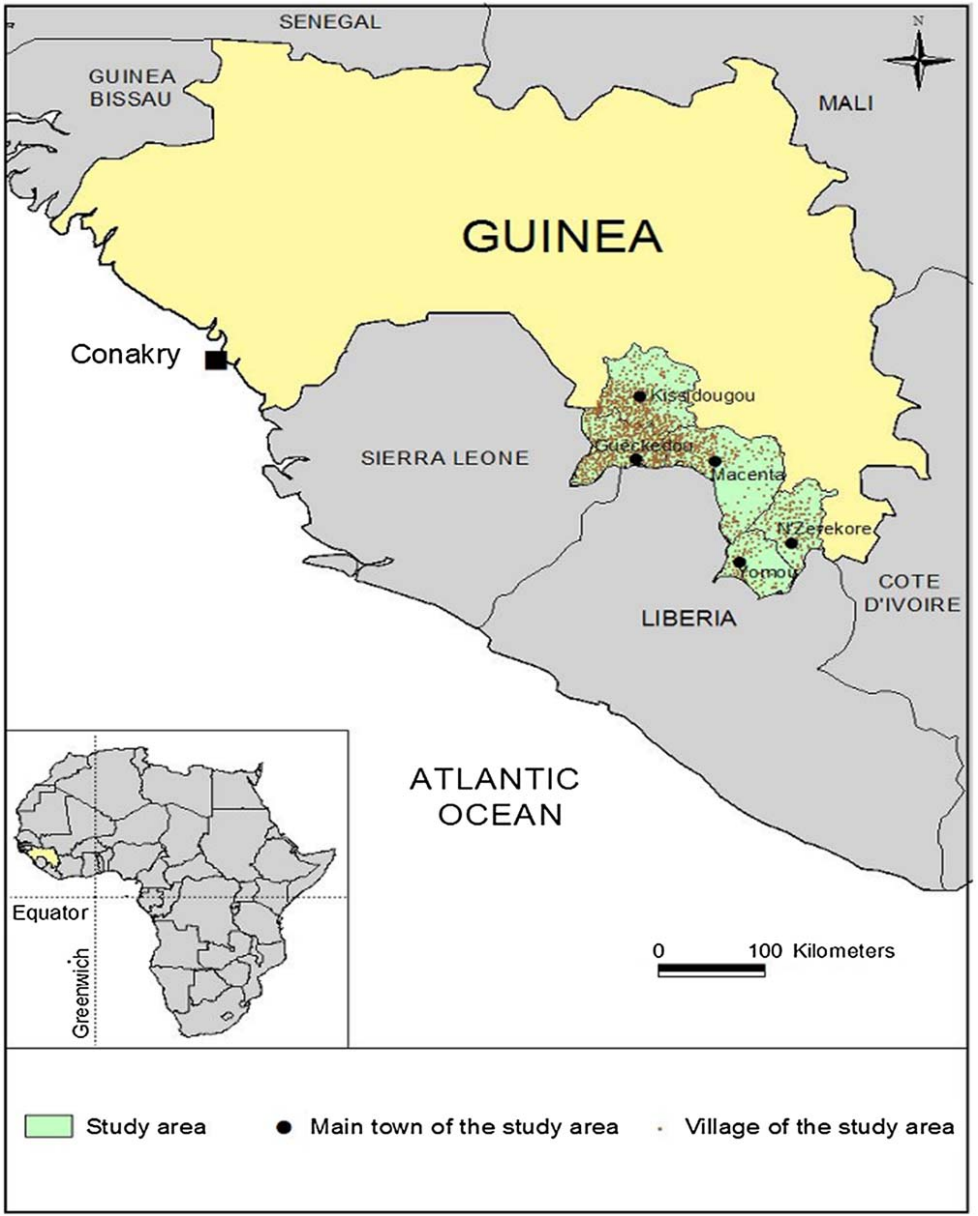
Location of the study area. The map displays the location of the study area, including the five main towns (Kissidougou, Guéckédou, Macenta, Yomou, and N’Zerekore) and all of the villages.

This is the case in the historical focus of forested Guinea, where the HAT epidemiological status remains unclear due to the scarcity of control activities conducted in recent decades as well as the absence of current information on tsetse fly distribution [19]. In order to implement suitable strategies that can reach the elimination goals in all forest areas of Guinea encompassing the historical foci of Kissidougou, Gueckedou, Macenta, Yomou and N’Zerekore, the National Control Programme of Human African Trypanosomiasis (NCPHAT) decided in 2012 to update the epidemiological situation of HAT in this broad area (2385 km^2^) with a population of 1420,530 inhabitants distributed in 1884 settlements (Fig. 1). In these large areas with probable low prevalence, exhaustive active screening is no longer suitable for updating the HAT situation. In this study, as an alternative, we implemented a novel approach based on geographical, entomological and epidemiological factors that can potentially influence disease distribution.

## Materials and methods

**Figure 2:**
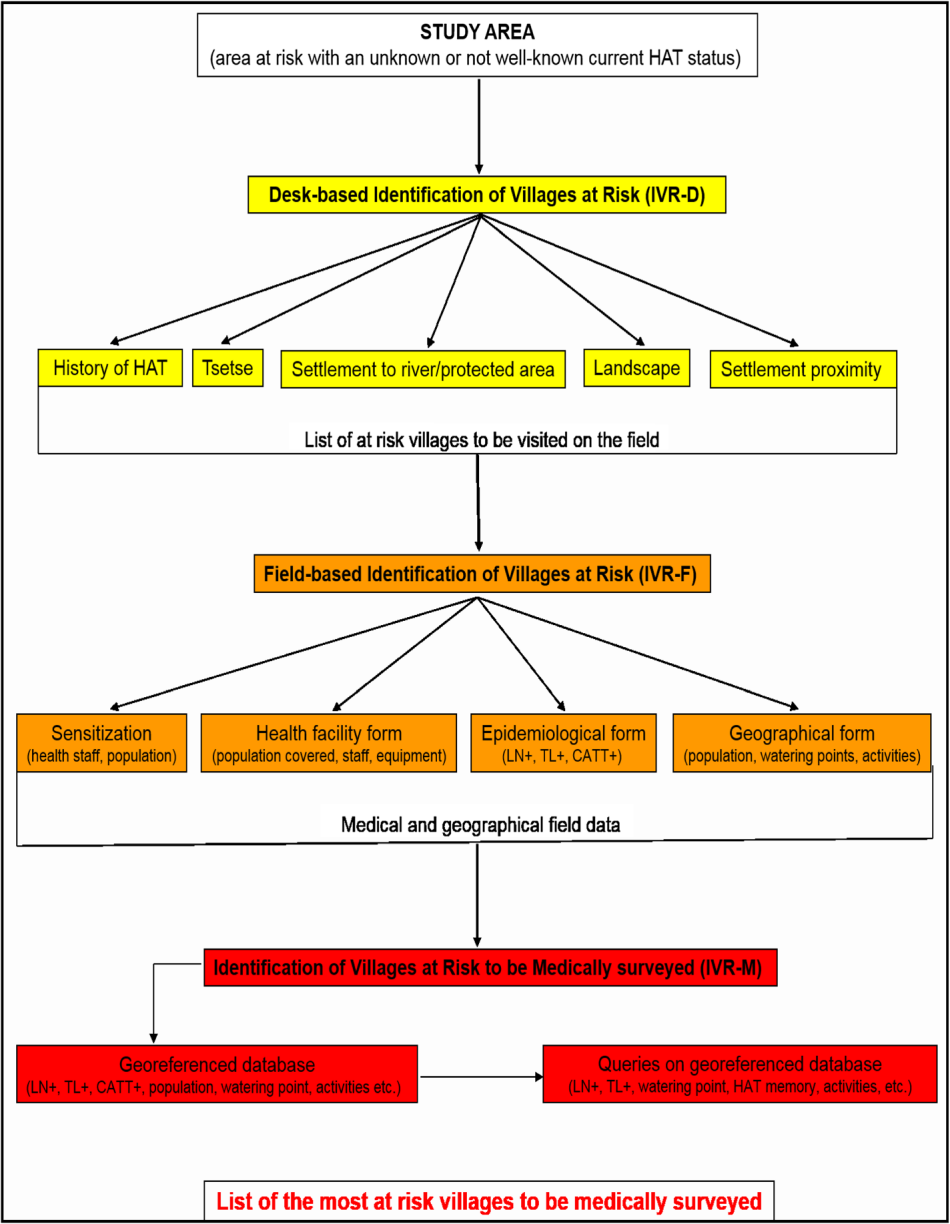
General procedure for IVR. The IVR strategy is structured into three main steps. The first step corresponds to IVR-D, which makes it possible to assemble an initial list of at-risk villages. The second step, IVR-F, is intended to collect field data about the main health facilities and villages on the initial list. The third step involves constructing a geo-referenced database to conduct queries, in order to select the most at-risk villages to be medically surveyed (IVR-M).

A methodology called Identification of Villages at Risk (IVR) was implemented in three sequential steps: (1) Desk-based Identification of Villages at Risk (IVR-D); (2) Field-based Identification of Villages at Risk (IVR-F); and (3) Identification of Villages at Risk by Medical survey (IVR-M) (Fig. 2). Then, exhaustive active screening, done by a mobile medical team who checked the entire population agreeing to be screened, was performed in the villages identified as the most at risk.

### Step 1: IVR-D

This step required one office-based person who has some knowledge of HAT epidemiology and tsetse ecology. This person reviewed HAT archives and worked with geographical tools (i.e., topographical maps and Google earth freeware) for 10 days.

#### History of HAT

Historical data on HAT distribution, occurrence and prevalence were collected from various archives (i.e., reports and maps). The locations of settlements formerly concerned by HAT were collected using old topographical maps. Data on the most recent HAT cases, detected after 2000, were obtained through the NCPHAT.

#### Tsetse species distribution

Data on tsetse distribution and species found in forested Guinea were recorded from the research literature.

#### Human settlement proximity to rivers and protected areas

The location of human settlements, hydrological networks and protected areas were determined from topographical maps and Google earth. The proximity of a settlement to a river or a protected area (e.g., national parks, classified forests) is a good indicator of human-tsetse contact. We considered that a distance of more than 4 km between a settlement and a river or a protected area does not constitute a risk.

#### State of landscape around villages

We used Google Earth to determinate the state of the landscape in the surroundings of the villages, looking for its suitability for tsetse.

#### At-risk human settlement proximity

When villages identified as “at risk”, thanks to the four previous layers described above, appear to be concentrated into a geographically limited area (i.e., less than 10 km between two villages), it can be assumed that the populations of these villages share the same working spaces and that the risk of HAT is quite similar. Consequently, villages with the formerly higher prevalence or with the most recent HAT cases diagnosed are selected first.

The spatial superimposition of the five information layers mentioned above made it possible to build a list of candidate villages at risk of HAT, that require a field visit in order to collect additional current epidemiological and geographical data. This list of at-risk villages derived from IVR-D is not restrictive, meaning that new villages can be included in the list following field observations (refer to the next step).

### Step 2: IVR-F

This step was performed over 12 days by a small mobile team (one geographer, one medical officer, one nurse, one driver) traveling from one village to the next by car. Due to accessibility, the season for implementing IVR-F must be taken into account. The team went into the field with the following material (an exhaustive list of material is given in the Supplementary Material 1):

- Documents to sensitise inhabitants to HAT (informative comic strips and posters);
- A box with tsetse fly specimens;
- GPS;
- All the material to perform the Card Agglutination Test for Trypanosomiasis (CATT/*Tbg*) [16], using a battery as a power source;
- Filter paper (Wattman^®^ No. 4) for blood sample collection in order to perform extemporaneous trypanolysis (TL) tests [2];
- A microscope using a battery as a power source for lymph node fluid examination;
- Three written documents: a “health facility form”, an “epidemiological form” and a “geographical form”.

**Figure 3:**
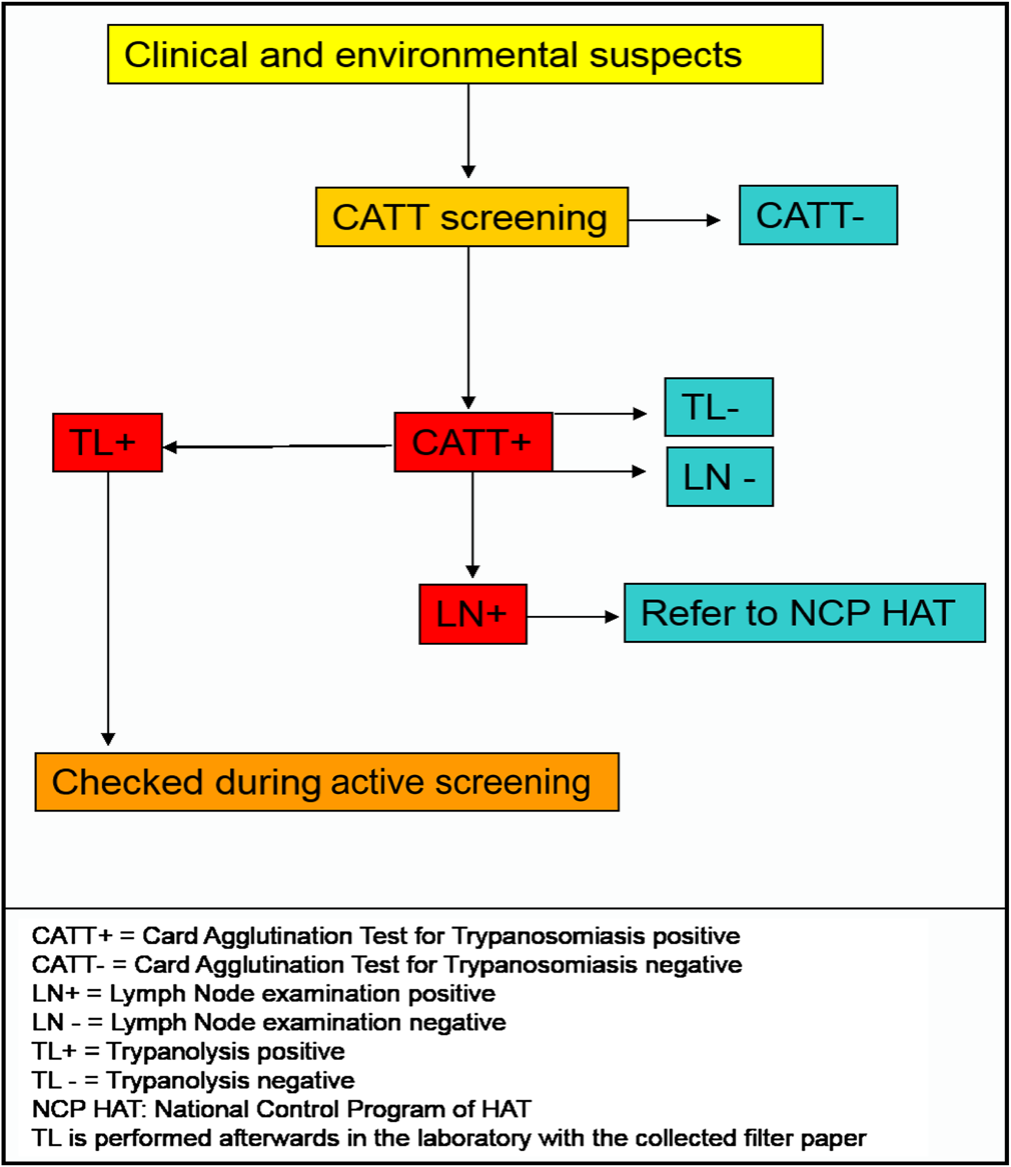
Medical diagnosis procedure during IVR. Clinical and environmental suspected cases are first screened by CATT. If positive, the lymph nodes of individuals are examined. CATT-positive individuals who are negative for LN and TL are considered HAT-negative. All TL-positive subjects must be checked during the active screening. CATT+ = Card Agglutination Test for Trypanosomiasis positive; CATT− = Card Agglutination Test for Trypanosomiasis negative; LN+ = Lymph Node examination positive; LN− = Lymph Node examination negative; TL+ = Trypanolysis positive; TL− = Trypanolysis negative; NCPHAT: National Control Programme of HAT. TL is performed afterwards in the laboratory with the collected filter paper.

The team explained to the central and local health staff the aim of the work and described the epidemiology of HAT. The various documents concerning HAT (i.e., informative comic strips and posters) were provided to the medical staff. The characteristics of the main health facilities (structure level, population covered, medical staff, availability of microscope, presence of lab technicians, etc.) were recorded on the “health facility form”. Then, the team met with the local population (especially community leaders and elders who are generally aware of HAT in historical foci), who were asked if they had any knowledge about HAT and tsetse flies (using a specimen) and if there were any potential suspected clinical cases in the village. Clinical suspicion of HAT was based on the following symptoms: recurrent fever and/or headache, presence of swollen cervical lymph nodes, and significant weight loss and neurological disorders. Subjects considered particularly at risk due to their daily activities reported to be at risk (such as fishing, rice cultivation, etc.) were also identified as “subject at environmental risk”. Then, all clinical and environmental suspects were serologically tested by the CATT/*Tbg* test [16]. A direct microscopic examination of Lymph Node (LN) aspirate was performed for all serologically positive subjects, whenever enlarged lymph nodes were observed. After checking that these subjects were not previously treated HAT cases, 50 μL of whole blood were sampled on filter paper (Wattman^®^ No. 4) to perform an immune trypanolysis test (TL), using the LiTat 1.3 variant antigenic type in a reference laboratory (Fig. 3). Since TL has in some studies shown very high specificity to detect the presence of specific antibodies against *Tbg* [11, 21], this test is increasingly used when looking at parasitologically unconfirmed CATT-seropositive subjects to identify those who are or were previously in contact with *Tbg* [11]. All epidemiological data were recorded on the “epidemiological form”.

Finally, the geographical coordinates of the visited villages were registered using a GPS device. Questions were also asked about the village population size, watering points (i.e., pumps, wells, and natural supply points), activities (i.e., type of cultivation, as well as fishing and hunting), animal breeding (especially pigs, which are known to be very attractive to tsetse and are suspected of carrying *Tbg* [12]), and population composition (natives, migrants or refugees and their geographical origin). All geographical data were registered on a “geographical form”.

### Step 3: IVR-M

This step was performed by the health geographer who was involved in IVR-D and IVR-F, and who built the database and performed queries at the office. The geographic coordinates of the visited settlements were downloaded from the GPS device to a computer using DNR Garmin software. Subsequently, the epidemiological and geographical datasets were organised into a geo-referenced database in an Excel file, with details on the primary health facilities, epidemiological status of the population screened, and the geography of the villages visited. Finally, the database was imported into Geographic Information System (GIS) software (ArcView) for mapping. Villages were first selected according to the level of HAT risk established by spatial queries performed on the epidemiological results, in descending order of importance: positive-LN subjects; positive-TL subjects, and high level of population memory regarding HAT. Subsequently, queries were made on geographical information, in descending order of importance: absence of pumps in the village; primary activities such as fishing, hunting, wood cutting, rice cultivation, rice-fish farming; presence of migrants/refugees coming from HAT endemic area; presence of pig breeding, and low economic level of the village. Analysing these parameters resulted in a list of the most at-risk villages. The number of villages selected for active screening can also depend on the available funds.

### Active screening and surveillance system

This step was performed by a classical HAT mobile medical team. First, all subjects were tested by CATT, which was performed on whole blood collected by finger prick (CATT-B). For CATT-B-positive subjects, blood was collected in heparinised tubes and a two-fold plasma dilution series in CATT buffer was performed to assess the end titration, i.e. the highest dilution still positive (CATT-P). All subjects displaying CATT-P ≥ 1/4 received parasitological examinations of the blood by direct examination of the LN and/or mini-Anion Exchange Centrifugation Technique (mAECT) [15]. Blood from CATT-B-positive subjects with negative parasitology results was sampled for extemporaneous highly specific TL using the LiTat 1.3 variant antigen type, as described above. The epidemiological data collected during IVR-F plus active screening (TL-positive subjects) and the characteristics of the health facilities visited (proximity with TL-positive subjects, health capacities) were considered for the selection of sentinel sites.

## Results

### Step 1: IVR-D

#### History of HAT

**Figure 4:**
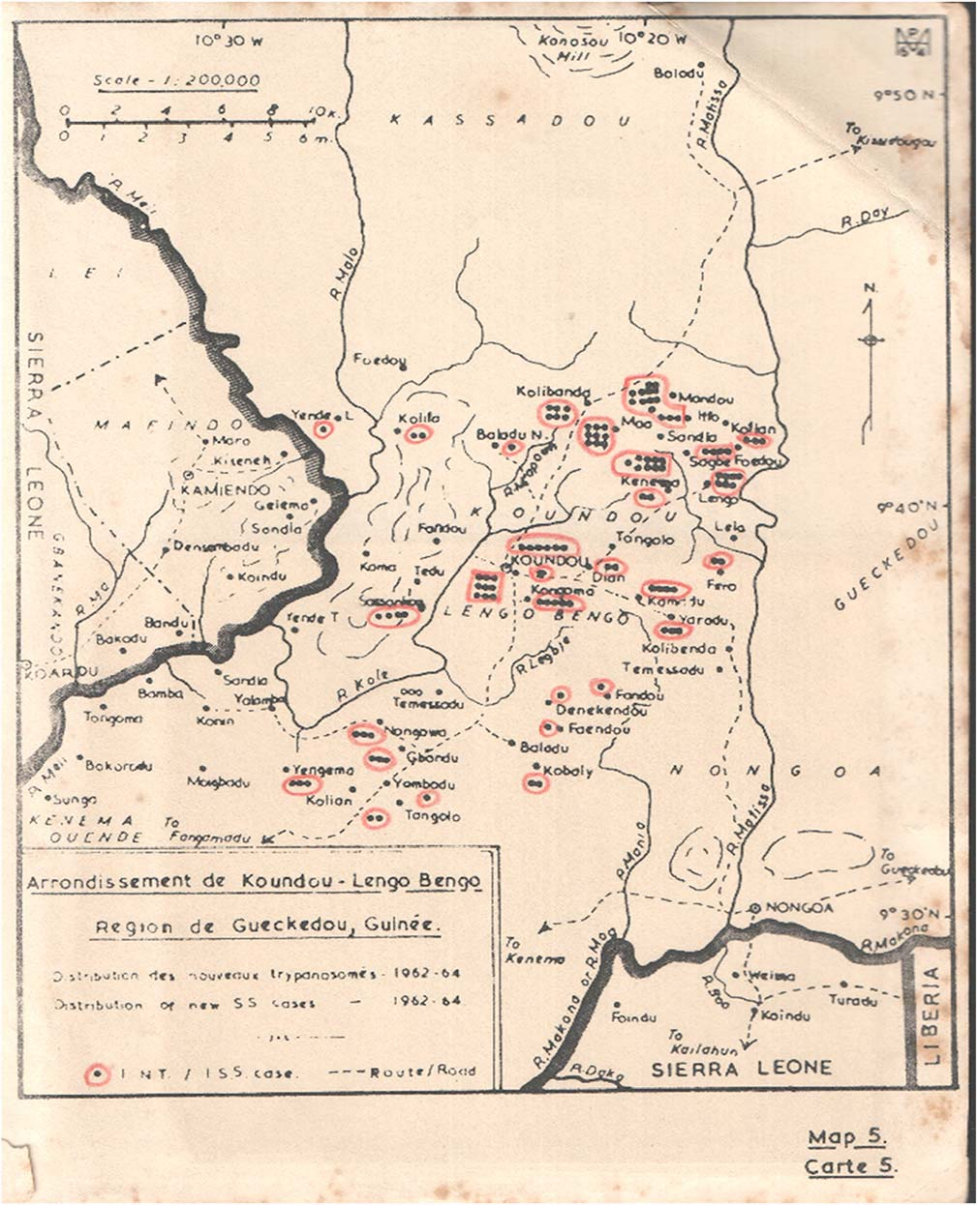
Location of HAT cases diagnosed from 1962 to 1964 in the Koundou Lengo Bengo focus (Gueckedou area). The map displays the distribution of HAT cases diagnosed from 1962 to 1964 in the Koundou Lengo Bengo focus, located in the Gueckedou area. This type of information is crucial in establishing the list of villages at risk of HAT to be visited in the field. Black dots with red circles represent the number of new sleeping sickness cases diagnosed between 1962 and 1964.

**Figure 5:**
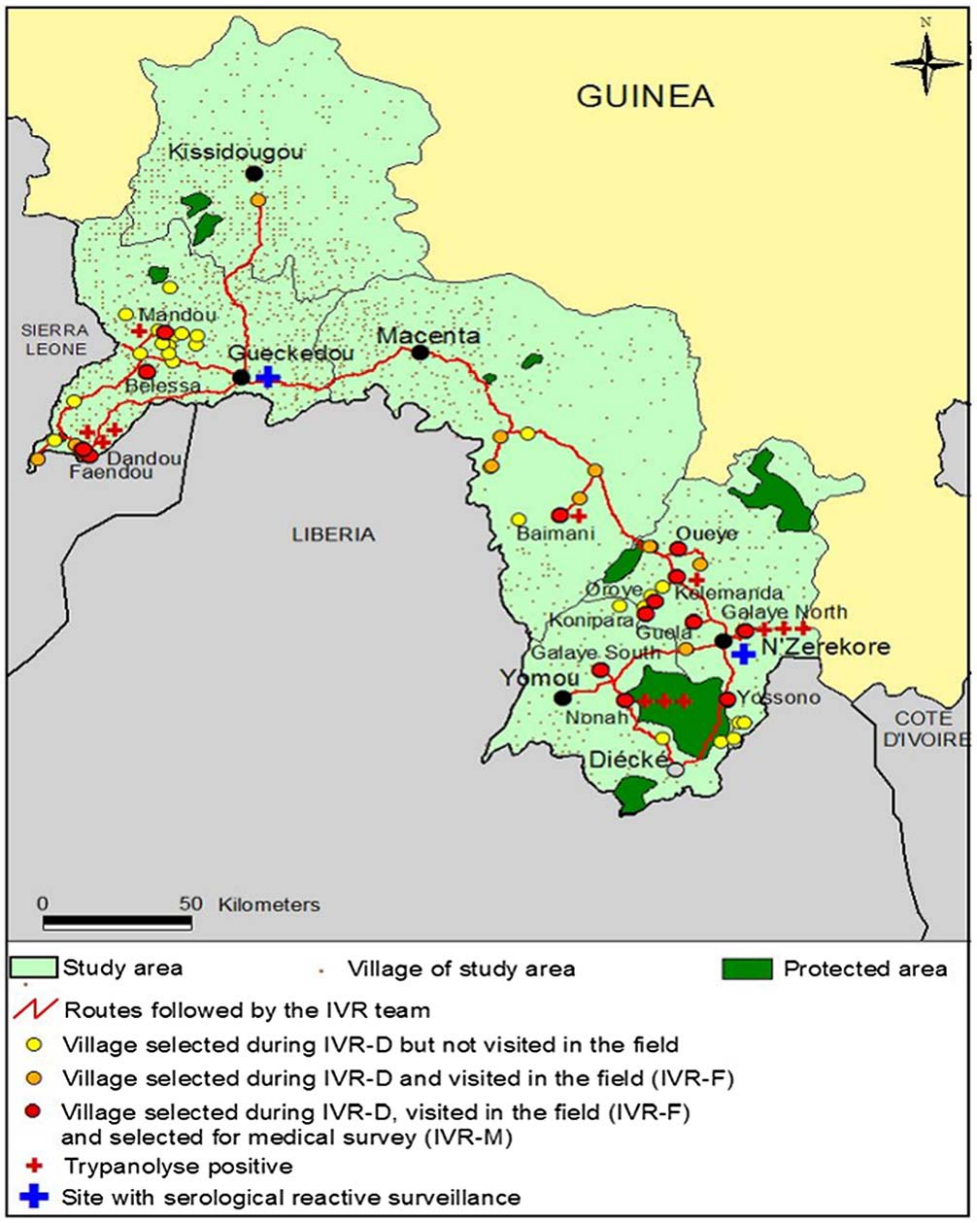
Villages identified during the IVR-D step, villages visited in the field (IVR-F), and villages selected for the active screening (IVR-M). The map displays the 49 villages selected during the IVR-D step, and the itinerary followed by the IVR team in the field to visit 24 of the villages. Fourteen of these villages were selected for the active screening, which are primarily located in the Gueckedou and N’Zerekore areas.

The history of HAT in the forest area of Guinea was primarily reviewed from the report of Hutchinson *et al.* [10]. This report helped us to localise the latest active HAT foci in the forest area of Guinea. Figure 4 displays the location of 111 HAT cases diagnosed in the Koundou Lengo Bengo focus (Gueckedou area) from 1962 to 1964. In this focus, three HAT cases were also reported in 2004 in the village of Belessa (NCPHAT information). To accurately localise all the villages quoted in the literature, we used topographical maps (1:200,000 scale) of Kissidougou, Gueckedou, Macenta, N’Zerekore and Tinsou that were made in the 1940s by the French Institut Géographique National (IGN). This first step led to the identification and localisation of 49 villages historically concerned by HAT (Table 1, Fig. 5).

**Table 1:** Names and geographical coordinates of the 49 villages identified as at risk of HAT.

ID	Name of settlement	Lat	Long	Visited	Reasons	Level of risk	Reasons	Active screening
*1*	*Bouye*	*9.122331*	−*10.094464*	*Yes*	*Proximity with river*	*Low*	*Low memory of HAT, pump, casava cultivation, horse breeding, no pig breeding, high economic level*	*No*
2	Kolibenda	8.616629	−10.324742	No	Landscape degradation	Not visited in the field	Not visited in the field	No
3	Moa	8.713916	−10.364047	No	Far from river and protected area	Not visited in the field	Not visited in the field	No
**4**	**Mandou**	**8.707567**	−**10.350263**	**Yes**	**High presence of HAT in the past, landscape conservation**	**High**	**TL+, high memory of HAT, no pump, rice cultivation, fishing, pig breeding, low economic level**	**Yes**
5	Kofian	8.695240	−10.321637	No	Proximity with Mandou	Not visited in the field	Not visited in the field	No
6	Lengo	8.666590	−10.336863	No	Landscape degradation	Not visited in the field	Not visited in the field	No
7	Temessadou	8.667514	−10.262054	No	Proximity with Mandou	Not visited in the field	Not visited in the field	No
8	Koendou	8.695980	−10.257788	No	Proximity with Mandou	Not visited in the field	Not visited in the field	No
9	Seoua	8.704113	−10.299909	No	Proximity with Mandou	Not visited in the field	Not visited in the field	No
10	Fero	8.639217	−10.336077	No	Landscape degradation	Not visited in the field	Not visited in the field	No
11	Kenema	8.670544	−10.352943	No	Proximity with Mandou	Not visited in the field	Not visited in the field	No
12	Koundou Lengo Bengo	8.640071	−10.415120	No	Landscape degradation	Not visited in the field	Not visited in the field	No
13	Singuedou	8.764007	−10.454232	No	Landscape degradation	Not visited in the field	Not visited in the field	No
14	Bolodou	8.849499	−10.333244	No	Landscape degradation	Not visited in the field	Not visited in the field	No
**15**	**Belessa**	**8.581038**	−**10.394384**	**Yes**	**Three HAT cases diagnosed in 2004, close to low ground, landscape conservation**	**Moderate**	**High memory of HAT, no pump, rice cultivation, pig breeding**	**Yes**
16	Fangamandou	8.490776	−10.592525	No	Inaccessibility	Not visited in the field	Not visited in the field	No
17	Kouloumba	8.367041	−10.647591	No	Proximity with Koundoutoh	Not visited in the field	Not visited in the field	No
*18*	*Koundoutoh*	*8.355026*	−*10.589004*	*Yes*	*Proximity with rivers, swamp area, proximity with Kailahun Sierra Leone HAT focus*	*Low*	*Low memory of HAT, pump, casava cultivation, no pig breeding*	*No*
*19*	*Kelema*	*8.308261*	−*10.691162*	*Yes*	*Proximity with rivers, landscape conservation, proximity with Kelema protected area*	*Low*	*Low memory of HAT, pump, casava cultivation, no pig breeding*	*No*
**20**	**Dandou**	**8.322826**	−**10.560335**	**Yes**	**Proximity with rivers, swamp area, proximity with Kailahun Sierra Leone HAT focus**	**High**	**TL+, Sierra Leone refugee**	**Yes**
**21**	**Faendou**	**8.339701**	−**10.574311**	**Yes**	**Proximity with rivers, swamp area, proximity with Kailahun Sierra Leone HAT focus**	**High**	**TL+, Sierra Leone refugee**	**Yes**
*22*	*Massadou*	*8.381913*	−*9.431443*	*Yes*	*Proximity with river, Proximity with protected area, proximity with Vonjaima Liberian HAT focus*	*Low*	*Low memory of HAT, pump, casava cultivation, no pig breeding*	*No*
*23*	*Sedimai*	*8.291126*	−*9.457742*	*Yes*	*Proximity with protected area, proximity with Vonjaima Liberian HAT focus*	*Low*	*Low memory of HAT, pump, casava cultivation, no pig breeding*	*No*
24	Zoubouroumai	8.392335	−9.357171	No	Proximity with Massadou	Not visited in the field	Not visited in the field	No
25	Soundedou	8.288638	−9.458559	No	Proximity with Sedimai	Not visited in the field	Not visited in the field	No
*26*	*Irie*	*8.276110*	−*9.174340*	*Yes*	*Located in low ground, proximity with Ziama protected area*	*Low*	*Low memory of HAT, pump, casava cultivation, no pig breeding*	*No*
*27*	*Boa*	*8.190635*	−*9.217781*	*Yes*	*Proximity with Ziama protected area, proximity with Baimani*	*Low*	*Low memory of HAT, pump, casava cultivation, no pig breeding*	*No*
**28**	**Baimani**	**8.134357**	−**9.272770**	**Yes**	**Proximity with Ziama protected area**	**Moderate**	**High memory of HAT, rice cultivation, pig breeding**	**Yes**
29	Gboda	8.121842	−9.380932	No	Proximity with Baimani	Not visited in the field	Not visited in the field	No
**30**	**Oroye**	**7.864713**	−**9.007785**	**Yes**	**Proximity with river, landscape conservation**	**Moderate**	**High memory of HAT, rice cultivation, pig breeding**	**Yes**
31	Kobela	7.884485	−9.023109	No	Proximity with Oroye	Not visited in the field	Not visited in the field	No
32	Neaye	7.912061	−8.988618	No	Proximity with Kelemanda	Not visited in the field	Not visited in the field	No
**33**	**Kelemanda**	**7.944325**	−**8.954135**	**Yes**	**Proximity to river, landscape conservation**	**High**	**TL+, rice cultivation, pig breeding**	**Yes**
34	Oulo	7.851371	−9.107593	No	Far from river and protected area	Not visited in the field	Not visited in the field	No
**35**	**Oueye**	**8.033632**	−**8.948995**	**Yes**	**High presence of HAT in the past, landscape conservation, close to low ground**	**Moderate**	**High memory of HAT, no pump, wood cutting, rice**−**fish farming, pig breeding, low economic level**	**Yes**
*36*	*Koule*	*8.036393*	−*9.021586*	*No*	*High presence of HAT in the past*	*Low*	*Low memory of HAT, pump, casava cultivation, no pig breeding*	*No*
**37**	**Guela**	**7.798113**	−**8.908240**	**Yes**	**High presence of HAT in the past, landscape conservation, proximity with river**	**Moderate**	**High memory of HAT, wood cutting, rice-fish farming, pig breeding, low economic level**	**Yes**
**38**	**Konipara**	**7.849861**	−**9.040703**	**Yes**	**High presence of HAT in the past, proximity with river, landscape conservation**	**Moderate**	**High memory of HAT, no pump, rice-fish farming, pig breeding, low economic level**	**Yes**
*39*	*Gbonoma*	*7.981428*	−*8.888323*	*Yes*	*Proximity with river, landscape conservation*	*Low*	*Low memory of HAT, pump, casava cultivation, no pig breeding*	*No*
*40*	*Kerema*	*7.716980*	−*8.927370*	*Yes*	*Landscape conservation, close to low ground*	*Low*	*Low memory of HAT, pump, casava cultivation, no pig breeding*	*No*
**41**	**Galaye North**	**7.773030**	−**8.770480**	**Yes**	**Close to lowground**	**Moderate**	**No pump, rice-fish farming, pig breeding, low economic level**	**Yes**
**42**	**Nonah**	**7.555770**	−**9.089550**	**Yes**	**Proximity with Diecke protected area**	**Moderate**	**No pump, rice-fish farming, pig breeding, Liberian refugee, low economic level**	**Yes**
**43**	**Yossono**	**7.552852**	−**8.815732**	**Yes**	**Proximity with Diecke protected area, close to lowground**	**Moderate**	**High memory of HAT, Liberian refugee, low economic level**	**Yes**
44	Kotonhui	7.435987	−8.989097	No	Far from river and protected area	Not visited in the field	Not visited in the field	No
**45**	**Galaye South**	**7.654670**	−**9.156030**	**Yes**	**Landscape conservation, close to low ground**	**Moderate**	**High memory of HAT, hunting, rice-fish farming, pig, Liberian refugee, low economic level**	**Yes**
46	Gerpa	7.427766	−8.831665	No	Inaccessibility	Not visited in the field	Not visited in the field	No
47	Douloupa	7.434890	−8.796662	No	Inaccessibility	Not visited in the field	Not visited in the field	No
48	Manaouen	7.484887	−8.783733	No	Inaccessibility	Not visited in the field	Not visited in the field	No
49	Beleton	7.485153	−8.769392	No	Inaccessibility	Not visited in the field	Not visited in the field	No

Black: Village selected during IVR-D but not visited in the field during IVR-F. Italic: Village selected during IVR-D and visited on the field during IVR-F. Bold: Village visited during IVR-D, visited in the field during IVR-F and selected for active screening.

#### Tsetse fly species distribution

Since very little information is available on current tsetse fly distribution in the forest area of Guinea, we relied on Ford and Katondo’s seminal study, which was conducted to identify tsetse distribution in this area (available at http://www.sleeping-sickness.ird.fr/cartes/cadre_carte.htm) [7]. These authors reported the tsetse species *G. palpalis*, *G. p. pallicera*, *G. fusca* and *G. nigrofusca* in this area. We also took into account information regarding the predicted distribution of tsetse flies in West Africa (available at http://www.fao.org/ag/againfo/programmes/en/paat/maps) [25]. According to this analysis, *G. palpalis* (the main HAT vector in West Africa) is distributed across the whole IVR intervention area, meaning that none of the 49 villages could be removed from the list by considering this information layer.

#### Settlement proximity to hydrological networks or well-protected areas

Settlements, hydrological networks and protected game areas were first identified using topographical maps (1:200,000 scale) of Kissidougou, Gueckedou, Macenta, N’Zerekore and Tinsou made in the 1940s by the French IGN. Through Google Earth we looked at the distance between the 49 villages and the river networks and protected areas. This analysis made it possible to remove three villages from the list of villages to be visited in the field during IVR-F: Moa, Oulo and Kotonhui (Table 1).

#### State of landscape around villages

In the mid-20th century, the population density of the Kissi ethnic group was approximately 37 inhabitants/km^2^, meaning that this is an old and important focus of human settlement in West Africa [5]. Nowadays, the density has increased to more than 100 inhabitants/km^2^ [1]. This increase in population density has modified the landscape in numerous places through such activities as clearing of vegetation for agriculture. The tsetse population (and therefore human-tsetse contact) has thus decreased in these areas, as a consequence of this destruction of favourable tsetse habitat. Accordingly, several villages previously affected by HAT are less at risk of *Tbg* transmission. As a result of this landscape degradation (which can be qualitatively observed on Google Earth), we decided to remove six villages (Kolibenda, Lengo, Fero, Koundou Lengo Bengo, Singuedou, Bolodou) from the list of the 46 villages remaining in the IVR-D list (Table 1).

#### At-risk settlement proximity

To investigate at-risk settlement proximity, we gave the priority to the villages that were most highly or most recently infected by HAT. For example, in the Koundou Lengo Bengo focus, only Mandou (highly infected in the past) and Belessa (more recently infected) were chosen for field investigations (Table 1, Fig. 5). Using this parameter for the whole forest area, 11 villages located less than 10 km from a village highly or recently infected, were removed from the IVR-D list (Kofian, Temessadou, Koendou, Seoua, Kenema, Kouloumba, Zoubouroumai, Soundedou, Gboda, Kobela, and Neaye). The 29 remaining villages were then selected for IVR-F (Table 1; Fig. 5).

### Step 2: IVR-F

Out of the 29 villages selected by IVR-D, 5 (Fangamandou, Gerpa, Douloupa, Manaouen, and Beleton) could not be visited due to poor roads in the area investigated and the associated time loss (Table 1). Thus, a total of 24 villages (40,459 inhabitants) were examined during IVR-F, representing 1.3% of the settlements at risk and 2.8% of the total population in the study area.

During IVR-F, we first met the medical staff of the main health facilities in Kissidougou, Guéckédou, Macenta, N’Zérékoré and Yomou to explain the objectives of the study and to collect data on these structures (structure level, population covered, medical staff) (Supplementary Material 2 “health facility form”). Next, we provided information to the local medical staff and the inhabitants of the 24 villages on how to identify clinical and environmental suspected cases, using HAT documents (i.e., posters or comic strips) and a display box with tsetse fly specimens. This step also enabled us to determine the level of historical HAT knowledge in the population, by listening to accounts by the chief and elders on former HAT cases in the villages, or mobile medical teams that used to screen the population for HAT. Based on both the knowledge of HAT symptoms by the local medical staff and the knowledge of the chief and elders of the villages, a sample of 222 clinical or environmental suspected cases were screened among the 24 villages and tested by CATT; 15 of these suspects were CATT-positive (6.7%) but LN-negative. Five of the 15 serological suspected cases tested positive for TL, indicating the presence of *Tbg*-specific antibodies in these individuals (Supplementary Material 2 “epidemiological form”). None of these subjects reported previous treatment for HAT. In addition to diagnostic activities, geographical indicators on the risk of human-tsetse contact were also recorded during the village visits. This included the absence of pumps in the village (thereby requiring the inhabitants to go to the river for water), as well as fishing, hunting, wood cutting, rice cultivation, and rice-fish farming activities. Details on the risk of *Tbg* presence/introduction were also recorded for pig breeding, the presence of migrants/refugees coming from HAT endemic areas, and the economic level of the village (Supplementary Material 1, “geographical form”).

### Step 3: IVR-M

Based on the collected IVR-D and IVR-F data, two areas appeared to be especially at risk of HAT: Gueckedou (including the villages of Mandou, Faendou and Dandou) and N’Zerekore (Kélémanda village), in which positive TL results suggested contact between *Tbg* and humans (Fig. 5). These four villages were classified as high-risk. Ten others villages displaying a moderate risk were also considered, taking into account the level of population memory on HAT and geographical results, i.e., factors favourable to human-tsetse contact (no pump in the village, activities as fishing, hunting, wood cutting, rice cultivation, and rice-fish farming) and factors favourable to the introduction/presence of *Tbg* (presence of migrants/refugees coming from HAT endemic areas, pig breeding, low economic level of the village; Table 1). The 10 remaining villages were classified as low-risk, based on epidemiological (no positive TL results) and geographical results (i.e., pumps in the village, low population memory of HAT, activities not favourable to human-tsetse contact, and no factors favourable to the introduction/presence of *Tbg*; Table 1). The 14 villages classified as high or moderate risk, comprising 11,236 inhabitants, were proposed for active screening (Fig. 5).

### Active screening and surveillance system

In December 2012, 4939 people were tested (population attendance of 44%), 55 of whom were CATT-P-positive (1.1%) with absence of enlarged lymph nodes. No HAT cases were diagnosed, although seven were TL-positive: three in Galaye North, three in Nonah, and one in Baimani (Fig. 5). None of the seven subjects were former HAT patients that had been previously treated. The five CATT-B and TL-positive subjects identified during IVR-F were also tested. These subjects were all positive for CATT-P and TL; however, no trypanosomes were detected by parasitological methods. According to the location of the 12 subjects positive to TL, and taking into account the capacities of health facilities, two new sentinel sites (Gueckedou and N’Zerekore) were selected for passive surveillance (Fig. 5).

## Discussion

All countries that want their elimination status validated by the WHO must show that they have implemented surveillance activities to clarify the current status of HAT in all at-risk areas. Exhaustive active screening alone is no longer suitable to obtain an overview of the HAT situation in historical foci or other broad areas at risk of HAT, due to the large surface areas that must be analysed, significant population sizes, and a general low prevalence of the disease, as illustrated here in the forest area of Guinea [19]. The complementary approach we describe here employs a geographically based methodology to help target active screening to those settlements most at risk, in order to update the local HAT situation. The IVR method may help to reach the objective of HAT elimination by focusing active screening on the most at risk villages, to assess the epidemiological situation of HAT in a defined focus, but also to establish an integrated HAT passive surveillance system and help to select the health facilities where it should be implemented.

Using this IVR methodology, we analysed an area with a population of 1420,530 potentially at-risk inhabitants located in 1884 settlements covering a surface of 2385 km^2^, which allowed us to identify for active screening the 14 most at-risk villages with a combined population of 11,236 inhabitants (i.e., 1.5% of the entire initial population). No HAT cases were diagnosed during the active screening or IVR, although 12 subjects tested positive for TL, suggesting past or current contact with *Tbg*. Unfortunately, these subjects could not be followed-up as recommended by the national diagnosis algorithm and their status could not be clarified. Furthermore, we cannot exclude the presence of HAT cases among the 56% of the population that was not tested during active screening. However, five out of the 222 people screened during the IVR were TL-positive (2.2%), and 7 out of the 4939 people screened during the exhaustive active screening were TL-positive (0.1%), showing the appropriateness of IVR in targeting individuals at high risk. Following these results, the Guinean NCPHAT decided to implement a passive surveillance system integrated into sentinel sites in the study area. Thanks to the data collected from health facilities during the IVR-F step, as well as the localisation of TL-positive subjects, hospitals in Gueckedou and N’Zerekore have been identified as sentinel sites.

According to the national procedures, TL-positive subjects who are parasitologically negative must be followed up until there is confirmation of *Tbg* (and its treatment) or serological negativation. Such subjects could represent individuals with long-lasting latent infections, as reported in active disease foci from Guinea and Côte d’Ivoire [11]. It should be noted that the final result of this activity is not the number of cases found, but rather the implementation of surveillance activities, which will result in detecting the presence or absence of HAT cases. We acknowledge the possibility that the IVR method may miss potential HAT cases if they are located in non-visited villages, since they would not have been identified as at-risk by IVR-D. In fact, since no medical activity was performed in any village that was not selected by IVR-D, the HAT status of these villages remains unknown and they cannot be compared to any of the villages selected as most at-risk by IVR. Here, this applies to the five villages identified as at risk during IVR-F that could not be visited due to poor roads. The IVR process described here for the Guinean forest with its three steps (IVR-D, IVR-F, and IVR-M) can be applied in different ecological contexts. According to the context, the criteria chosen to determine the risks (symptoms, activities, etc.) will have to be adapted, and the material used could be changed (for example by using Rapid Diagnostic Tests for HAT instead of CATT). The results of similar exercises already performed in different settings (Senegal, Bissau Guinea, Niger, Chad, data not published yet), should allow us to identify the strong points, contributions, weaknesses and challenges of this methodology, and will probably help to develop a standardised protocol.

Keeping in mind these parameters, in West Africa, IVR can be applied to all of the historical foci in Gambia and Liberia, and some of the historical foci in Sierra Leone, Côte d’Ivoire, Guinea and Nigeria [4]. It can also be applied in regions characterised by landscape change due to immigration from endemic areas, such as the south western part of Côte d’Ivoire [14]. There are also known foci in Central Africa in which the intensity of transmission has not been clearly quantified, due to difficult topography (Democratic Republic of Congo) or safety constraints (some areas of Nigeria, the Central African Republic, and the Democratic Republic of Congo) [3, 23]. We acknowledge that IVR cannot be applied in known foci in which the transmission intensity has not been clearly quantified due to safety constraints. However, once such areas are secure, IVR will be a useful tool for updating the HAT situation. The method presented here, while pertinent to our study area, can also be followed as a protocol for other such areas. Although IVR was developed in the framework of HAT elimination, in terms of integrating disease surveillance, IVR could possibly be applied to other Neglected Tropical Diseases (onchocerciasis, Buruli ulcer, etc.) that affect the same populations as HAT.

In conclusion, the results of IVR combined with active screening allowed us to implement an adapted control strategy for the sustainable elimination of HAT in the historical focus of forested Guinea. The WHO and the international community have recently expressed their ambition to eliminate sleeping sickness, and the IVR method is a practical and timely approach to contribute to this goal. In the context of eliminating HAT, active screening must be preceded by geographically oriented activities such as IVR, which will permit a thorough assessment of the HAT situation in large areas with little or no recent data. Importantly, IVR could be performed in any *gambiense* foci where it is needed, thereby contributing to achieving this objective of sustainable HAT elimination.

## Supplementary materials

Supplementary material 1 Exhaustive list of the material needed to implement IVR in the Guinean forest. Supplementary material 2 The file provides the results collected through the “health facility form”, the “epidemiological form” and the “geographical form”. Supplementary material is available at https://www.parasite-journal.org/10.1051/parasite/2019061/olm.
