# Supplementary material for: Sleeping sickness in the historical focus of forested Guinea: update using a geographically based method
Source: Parasite. 2019 Oct 10;26:61. doi: 10.1051/parasite/2019061 (PMC6785972; doi:10.1051/parasite/2019061)
Supplement: Supplementary material 1 — Exhaustive list of the material needed to implement IVR in the Guinean forest. [file parasite-26-61-s1.docx]

**Supplementary material 1.** Exhaustive list of material needed.

Sensitization:

- Cartoon on HAT describing HAT symptoms, tsetse ecology and activities at risk (FAC/OMS, 1997),

- Posters describing HAT symptoms (NCPHAT/Sanofi, 2010).

Entomological:

- A box with tsetse fly specimens.

Geographical:

- Global Positioning System,

- Battery,

- Topographical maps,

- Camera,

- “Geographical form”.

Medical:

- CATT/*Tbg* Agitator,

- Battery,

- CATT/*Tbg* reagents and accessories,

- Gloves,

- Cotton,

- 90°C alcohol,

- Blood lancets,

- Heparinized capillary tubes,

- Filter paper (Wattman® no. 4),

- 200 µl graduated pipette with corresponding tips,

- Microscope using a battery as a power source,

- Syringes,

- Microscope slides and coverslips,

- Trash bags,

- “Epidemiological form”,

- “Health facilities form”.
