# Supplementary material for: Sleeping sickness in the historical focus of forested Guinea: update using a geographically based method
Source: Parasite. 2019 Oct 10;26:61. doi: 10.1051/parasite/2019061 (PMC6785972; doi:10.1051/parasite/2019061)
Supplement: Supplementary material 2 — The file provides the results collected through the “health facility form”, the “epidemiological form” and the “geographical form”. [file parasite-26-61-s2.docx]

**Supplementary Material 2.** Forms.

“health facility form”.

| **Town** | **Lat** | **Long** | **Structure level** | **PopC** | **SC** | **TC (%)** | **Staff** | **Doctor** | **NS** | **LT** | **TS** | **Equipment** |
| --- | --- | --- | --- | --- | --- | --- | --- | --- | --- | --- | --- | --- |
| Kissidougou | 9,188197 | -10,097926 | Prefectoral hospital | 273 822 | 40 HP, 17 HC, 1 HP | 55 | 50 | 16 | 31 | 3 | 216 | 2 microscopes,  1 centrifuge |
| Gueckedou | 8,563056 | -10,133478 | Prefectoral hospital | 297 544 | 25 HP, 13 HC, 1 MDC, 1 HP | 52 | 64 | 19 | 42 | 3 | 245 | 3 microscopes,  2 centrifuges |
| Macenta | 8,542874 | -9,469224 | Prefectoral hospital | 296 050 | 35 HP, 17 HC, 1 HP | 48 | 46 | 15 | 28 | 3 | 223 | 2 microscopes,  1 centrifuge |
| N'Zerekore | 7,752209 | -8,811322 | Regional hospital | 381 653 | 39 HP, 16 HC, 1 RH | 45 | 85 | 25 | 54 | 6 | 321 | 4 microscopes,  3 centrifuges |
| Yomou | 7,565996 | -9,261606 | Prefectoral hospital | 171 461 | 14 HP, 17 HC, 1 HP | 60 | 35 | 9 | 24 | 2 | 189 | 1 microscope,  1 centrifuge |

**Legend.**

| **PopC** | Population covered |
| --- | --- |
| **SC** | Number of structures covered |
| **HP** | Health Post |
| **HC** | Health Center |
| **MDC** | Medical District Center |
| **PH** | Prefectoral Hospital |
| **RH** | Regional Hospital |
| **TC** | Annual Consultative rate |
| **Staff** | Number of medical staff |
| **NS** | Nurse superior |
| **LT** | Laboratory technician |
| **TS** | Annual number of thick smear |
| **Equipment** | Number of microscope, centrifuge |

“epidemiological form”.

| **Settlement** | **Type** | **Lat** | **Long** | **LMHAT** | **CATT** | **CATT+** | **LN** | **LN+** | **TL+** |
| --- | --- | --- | --- | --- | --- | --- | --- | --- | --- |
| Bouye | Village | 9,122331 | -10,094464 | Low | 13 | 2 | 0 | 0 | 0 |
| Mandou | Hamlet | 8,707567 | -10,350263 | High | 32 | 2 | 0 | 0 | 1 |
| Belessa | Hamlet | 8,581038 | -10,394384 | High | 16 | 1 | 0 | 0 | 0 |
| Koundoutoh | Hamlet | 8,355026 | -10,589004 | Low | 10 | 0 | 0 | 0 | 0 |
| Kelema | Hamlet | 8,308261 | -10,691162 | Low | 17 | 0 | 0 | 0 | 0 |
| Dandou | Hamlet | 8,322826 | -10,560335 | Low | 17 | 3 | 0 | 0 | 2 |
| Faendou | Hamlet | 8,339701 | -10,574311 | High | 11 | 1 | 0 | 0 | 1 |
| Massadou | Village | 8,381913 | -9,431443 | Low | 1 | 0 | 0 | 0 | 0 |
| Sedimai | Village | 8,291126 | -9,457742 | Low | 16 | 0 | 0 | 0 | 0 |
| Irie | Town | 8,276110 | -9,174340 | Low | 5 | 0 | 0 | 0 | 0 |
| Boa | Town | 8,190635 | -9,217781 | Low | 12 | 0 | 0 | 0 | 0 |
| Baimani | Village | 8,134357 | -9,272770 | High | 16 | 1 | 0 | 0 | 0 |
| Oroye | Village | 7,864713 | -9,007785 | High | 11 | 2 | 0 | 0 | 0 |
| Kelemanda | Village | 7,944325 | -8,954135 | Low | 12 | 2 | 0 | 0 | 1 |
| Oueye | Village | 8,033632 | -8,948995 | High | 0 | 0 | 0 | 0 | 0 |
| Koule | Village | 8,036393 | -9,021586 | Low | 4 | 0 | 0 | 0 | 0 |
| Guela | Village | 7,798113 | -8,908240 | High | 0 | 0 | 0 | 0 | 0 |
| Konipara | Village | 7,849861 | -9,040703 | High | 0 | 0 | 0 | 0 | 0 |
| Gbonoma | Village | 7,981428 | -8,888323 | Low | 3 | 0 | 0 | 0 | 0 |
| Kerema | Town | 7,716980 | -8,927370 | Low | 2 | 0 | 0 | 0 | 0 |
| Galaye Nord | Village | 7,773030 | -8,770480 | High | 1 | 0 | 0 | 0 | 0 |
| Nonah | Village | 7,555770 | -9,089550 | High | 2 | 0 | 0 | 0 | 0 |
| Yossono | Village | 7,552852 | -8,815732 | High | 8 | 1 | 0 | 0 | 0 |
| Galaye Sud | Village | 7,654670 | -9,156030 | High | 13 | 0 | 0 | 0 | 0 |

**Legend.**

| **LMHAT** | Level of Memory of HAT |
| --- | --- |
| **CATT** | Screened with CATT |
| **CATT+** | CATT positive |
| **LN** | Lymph Nodes screened |
| **LN+** | Lymph Nodes positive |
| **TL+** | Trypanolyse positive |

“geographical form”.

| **Settlement** | **Lat** | **Long** | **Pop** | **Pumps** | **Wells** | **NWP** | **Activities** | **Breeding** | **MRO** | **ELV** |
| --- | --- | --- | --- | --- | --- | --- | --- | --- | --- | --- |
| Bouye | 9,122331 | -10,094464 | 1000 | 3 | 3 | 1 | Casava cultivation, Maize cultivation | Horse | No | High |
| Mandou | 8,707567 | -10,350263 | 200 | 0 | 2 | 1 | Rice cultivation, Fishing | Pig | No | Low |
| Belessa | 8,581038 | -10,394384 | 300 | 0 | 3 | 2 | Rice cultivation, Fishing | Pig | No | Low |
| Koundoutoh | 8,355026 | -10,589004 | 350 | 1 | 3 | 1 | Casava cultivation | Cattle | Sierra Leone | Middle |
| Kelema | 8,308261 | -10,691162 | 200 | 1 | 2 | 1 | Casava cultivation | No | Sierra Leone | Middle |
| Dandou | 8,322826 | -10,560335 | 450 | 0 | 4 | 3 | Rice cultivation, Palm trees, Fishing | Pig | Sierra Leone | Low |
| Faendou | 8,339701 | -10,574311 | 300 | 0 | 2 | 2 | Rice cultivation, Palm trees, Fishing | Pig | Sierra Leone | Low |
| Massadou | 8,381913 | -9,431443 | 1500 | 1 | 2 | 1 | Casava cultivation, Palm trees | No | Liberia | Middle |
| Sedimai | 8,291126 | -9,457742 | 2000 | 2 | 3 | 0 | Casava cultivation, Maize cultivation | Cattle | Liberia | Middle |
| Irie | 8,276110 | -9,174340 | 8500 | 5 | 8 | 0 | Casava cultivation, Maize cultivation | Cattle | No | High |
| Boa | 8,190635 | -9,217781 | 5743 | 3 | 7 | 0 | Casava cultivation, Palm trees | Cattle | No | High |
| Baimani | 8,134357 | -9,272770 | 800 | 1 | 4 | 0 | Casava cultivation, Rice cultivation, Hunting | Pig | No | Middle |
| Oroye | 7,864713 | -9,007785 | 986 | 1 | 5 | 2 | Rice cultivation | Pig | No | Middle |
| Kelemanda | 7,944325 | -8,954135 | 1981 | 2 | 5 | 2 | Rice cultivation | Pig | No | Middle |
| Oueye | 8,033632 | -8,948995 | 1500 | 0 | 3 | 3 | Rice-fish farming, Palm trees | Pig | No | Low |
| Koule | 8,036393 | -9,021586 | 1000 | 1 | 2 | 0 | Casava cultivation | No | No | Middle |
| Guela | 7,798113 | -8,908240 | 1500 | 1 | 3 | 3 | Rice-fish farming, Palm trees | Pig | No | Low |
| Konipara | 7,849861 | -9,040703 | 1000 | 0 | 2 | 2 | Rice-fish farming, Palm trees | Pig | No | Low |
| Gbonoma | 7,981428 | -8,888323 | 680 | 1 | 2 | 1 | Casava cultivation, Maize | No | No | High |
| Kerema | 7,716980 | -8,927370 | 7000 | 4 | 7 | 0 | Casava cultivation, Palm trees | Cattle | No | High |
| Galaye North | 7,773030 | -8,770480 | 500 | 0 | 2 | 1 | Rice-fish farming, Palm trees | Pig | No | Low |
| Nonah | 7,555770 | -9,089550 | 622 | 0 | 3 | 1 | Rice-fish farming, Palm trees, Hunting | Pig | Liberia | Low |
| Yossono | 7,552852 | -8,815732 | 600 | 1 | 3 | 2 | Rice-fish farming, Palm trees, Casava cultivation, Hunting | Pig | Liberia | Low |
| Galaye South | 7,654670 | -9,156030 | 1747 | 2 | 5 | 2 | Rice-fish farming, Palm trees, Hunting | Pig | Liberia | Low |

**Legend.**

| **NWP** | Natural Watering Point |
| --- | --- |
| **MRO** | Migrants Refugees Origins |
| **ELV** | Economic Level of the Village |
